# Salivary Calculus

**Published:** 1875-01

**Authors:** S. James A. Salter


### ARTICLE V.

### *Salivary Calculus.*

BY S. JAMES A. SALTER, ESQ , M. B., F. R. S., &C.

I have been making some investigations relative to salivary calculus, and in these I have been assisted by my friend and colleague, Dr. Stevenson, who analysed several specimens for me.

I have thought it might be desirable to place the results on record.

Salivary calculus exists in two distinct conditions—as *Tartar* on the teeth, and *Stones* within the ducts of the salivary glands.

Tartar differs a good deal in different specimens as to its physical and somewhat as to chemical characters. It is deposited from the compound fluids of the mouth, and it has, entangled in it, a certain amount of extraneous matter, such as shed epithelium and a notable quantity of the fibres of the mouth-fungus, *Leptothrix buccalis.*

The calcareous *Stones* that are found in the salivary ducts are necessarily the produce alone of the secretion of the glands in whose ducts they are formed.

Between tartar, in its several varieties, and the duct-stones there appears to be one constant chemical difference, namely that the latter contain a much larger amount of *carbonate* of lime.

Dr. Stevenson's analysis of tartar have been made with mixed specimens, soft tartar taken from molar teeth, and hard masses removed from the lower incisors.

MIXED SPECIMENS.

| | |
|---|---|
| Phosphoric acid | 29.72 |
| Lime | 35 70 |
| Magnesia | 6.22 |
| Organic matter | 20.27 |
| Water | 7.33 |
| Carbonic acid, a trace. | |
| | 90.24 |

SOFT TARTAR FROM MOLARS.

| | |
|---|---|
| Phosphate of lime with a little carbonate and a trace of fluoride | 77.21 |
| Phosphate of magnesia | 1.31 |
| Organic matter | 16.33 |
| Water | 5.15 |
| | 100.00 |

HARD TARTAR FROM LOWER INCISORS.

| | |
|---|---|
| Phosphate of lime, with a little carbonate and some fluoride | 81.18 |
| Phosphate of magnesia | 1.31 |
| Organic matter and water | 17 51 |
| | 100.00 |

I believe a considerable portion of the organic matter in tartar consists of Leptothrix fibres that have become encrusted and covered in by the calcareous deposit. Indeed I think it highly probable that the meshes of the fungus fibres may contribute to and facilitate the deposition and solidification of the lime salts.

If tartar is dissolved by hydrochloric acids numbers of minute threads are seen remaining when the specimen is examined by high powers of the microscope; and I have found that these so persistently resisted the solvent action of chemical reagents that I was disposed to think they were silicic in their nature. Dr. Stevenson, however, failed to discover a trace of silex in any of the specimens he analyzed.

It is said that the tartar formed on the molar teeth and supposed to be derived by the parotid glands contains more carbonate of lime than that deposited elsewhere. Dr. Stevenson's analyses did not bear out this idea.

The salivary duct-stones, as I have observed, contain much more carbonate of lime than the tartar on the teeth.

Some years ago Dr. Taylor, of Guy's Hospital, analyzed several of these concretions, and the following was the general result of a number of museum specimens, without distinctions as to the glands that furnished them.

| | |
|---|---|
| Animal matter | 25 |
| Phosphate of lime | 55 |
| Carbonate of lime | 15 |
| Carbonate of magnesia | 1 |
| Oxide of iron | 2 |
| Loss | 2 |
| | 100 |

The fact that the calculi were probably derived from different glands might be presumed to rob the analysis of much of its interest, as the secretion of the different salivary glands is not the same. Dr. Taylor, however, analyzed one large duct calculus, which certainly came from the submaxillary gland, and with nearly the same results, as shown below:

| | |
|---|---|
| Animal matter, salivary mucus containing nitrogen and sulphur - - - - - - - - - | 32.00 |
| Phosphate of lime, with traces of phosphate of magnesia | 55.40 |
| Carbonate of lime - - - - - - - - | 13.60 |
| | 100.00 |

The history of the latter calculus is interesting, and I cannot do better than conclude this brief communication by narrating it and an account of the patient from whom it was obtained.

Mrs H—, æt. 59, of healthy constitution, was seized on the 14th of February, 1874, with very severe pain at the base of the tongue and on the left side of the jaw in the situation of the submaxillary gland; great swelling took place in these parts during the day, and the gland was very hard, continuing to get rapidily worse for three days.

At this time Mr. F—(a general practitioner) was consulted. By making one or two small openings by the side of the frænum linguæ he suceeded in extracting three small calculi about the size of a goose shot. The pain abated for a short time after their extraction, but became worse towards evening. On the following night, leeches were applied to the side of the chin, as the size of the tongue and floor of the mouth, especially at the back part, almost obstructed the pharynx, and the breathing became very laborious. About an hour after the applications of the leeches an abscess broke near the entrance of the submaxillary duct, which gave great relief both as to pain and breathing. Although the swelling from this time gradually subsided, severe pain continued at intervals, the abscess appeared alternately to gather and break, the pain being alleviated after each discharge, whilst the submaxiliary gland remained very hard.

Matters continued in this state for above three months. With a bent probe a hard substance could be distinctly felt about one-third of an inch from the opening, and with a small pair of bent forceps little pieces could be broken off, but it was found impossible to remove a large piece. As the patient was not permanently relieved by the extraction of such small fragments, and it being probable that a large calculus was situated deeply in the duct, an incision was made about an inch and a half long by the side of the tongue so as to extend some distance before and behind the opening; a pair of dressing forceps was then inserted, and after a little trouble the calculus was removed; no particular hemorrhage occurred, but the pain was very great. A piece of lint was left in the wound, which healed completely in about a month without a bad symptom. The tongue gradually recovered its proper position; the submaxillary gland also, which had been previously almost of scirrhus hardness, slowly became soft and natural. The recovery was complete.

The calculus was nearly globular and the size of a small marble. It weighed fourteen grains.—*British Journal of Dental Science.*

---

